# Effectiveness of Sorafenib in Hepatic Hemangioma

**DOI:** 10.1200/JGO.2016.008573

**Published:** 2017-05-09

**Authors:** Prateek Tiwari, Vandana Mahajan, Kanchan Muhrerkar, Bhanu Jayanand Sunil, Ayloor Ramakrishnan, Trivadi Ganesan

**Affiliations:** **All authors:** Cancer Institute (WIA), Adyar, Chennai, India.

## INTRODUCTION

Hemangiomas are the most common benign tumors of the liver, with a frequency of 0.4% to 7.3%.^1^ Small liver hemangiomas (< 4 cm) are generally asymptomatic and can be observed without any chance of malignant transformation or complications. Hemangiomas are defined as giant if their size exceeds 4 cm.^2^ The most common site is the right lobe of the liver (subcapsular region). Small, asymptomatic hemangiomas do not show changes during long-term follow-up; hence they can be observed without any treatment.^3^ Giant liver hemangiomas are symptomatic and patients present with mild pain, abdominal mass, abdominal fullness and rarely with jaundice, consumptive coagulopathy (Kasabach-Merritt syndrome), or intra-abdominal bleeding as the result of rupture. Congestive heart failure and intraperitoneal bleeding can sometimes be fatal.^4^ Surgical resection is the only treatment option for symptomatic hemangiomas. Diagnosis is not easy to obtain because percutaneous biopsy is risky and the role of other treatment options such as steroids, hepatic artery ligation, and radiotherapy is controversial.^5^

Herein, we report an unusual case of a giant symptomatic hepatic hemangioma, which was initially diagnosed as an inoperable hepatocellular carcinoma (HCC) and treated with an oral tyrosine kinase inhibitor (ie, sorafenib).

## CASE REPORT

A 65-year-old woman visited our outpatient department with a complaint of weight loss for 1 year. She was in good general condition and had an Eastern Cooperative Oncology Group performance status of 1. Abdominal examination revealed an enlarged liver that was palpable 6 cm below the right costal margin; it was not tender and no other mass was felt. A complete hemogram as well as liver and renal function tests were normal. The coagulation profile was normal. Levels of carcinoembryonic antigen (1.94 ng/mL) and alpha-fetoprotein (2.13 ng/mL) were within the normal range.

**Fig 1:**
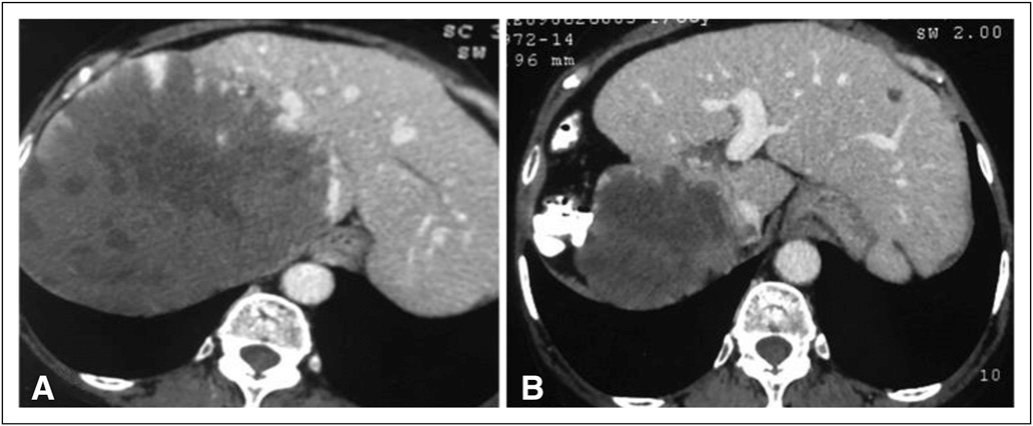
(A) Contrast computed tomography scan of the abdomen of a 65-year-old woman showing a large heterogeneous mass in the right lobe of the liver. (B) Significant regression of the liver mass with compensatory hypertrophy of the left lobe of the liver.

Contrast computed tomography (CT) of the abdomen at presentation (Fig 1A) revealed a large heterogeneous mass (18 × 12 × 12 cm) involving the entire right hepatic lobe (anterior and posterior segments), displacing hepatic veins and the inferior vena cava. The mass showed multiple nonenhancing areas, which were suggestive of necrosis. A tiny calcification was found in the periphery of the lesion. Two small hypodense areas were found on the left hepatic lobe, which was suggestive of simple cysts. The mass showed early arterial enhancement, which is usually noticed in vascular lesions.

Ultrasound-guided fine-needle aspiration cytology of the liver was done twice and both times it showed blood and tiny fragments of the liver with few dilated vascular channels. Because biopsy was unsuccessful, a provisional diagnosis of HCC was established on the basis of radiologic findings.

The mass was considered inoperable because it involved all three hepatic veins and the right branch of the portal vein. Therefore, with a presumptive diagnosis of HCC, the decision was made to start sorafenib at a dosage of 800 mg per day. The patient developed grade 2 hand-foot syndrome, which was observed approximately 1 month after administration of sorafenib and subsequently settled down after tapering and adjusting the dosage to 200 mg per day. This was the dose that the patient could tolerate. Sequential ultrasonography of the abdomen after 5 months of treatment with sorafenib revealed a 40% size reduction; hence the same dose of sorafenib was maintained.

A repeat CT scan after 18 months of regular treatment with sorafenib revealed remarkable tumor size reduction with atrophy of the right lobe of the liver (Fig 1B). The tumor measured 10.8 × 9.8 cm. There was compensatory hypertrophy of the left lobe of the liver. The left lobe cyst appeared static. Because the CT scan showed that the tumor had undergone significant reduction and was operable, a right hepatectomy was planned for the patient.

A positron emission tomography–CT scan was performed, which confirmed an irregular lobulated mass in the right lobe of the liver involving segments 5, 6, and 7 (size: 8.1 cm [anteroposteriorly] × 9.2 cm [width] ×10.8 cm [craniocaudally]), with increased metabolic activity (standard uptake value, 3.2) and a tiny non–[^18^F]fluorodeoxyglucose-avid simple cyst in the left lobe of the liver. There was no uptake elsewhere in the body.

The general health of the patient was fit on the basis of other routine investigations required for the surgery. Right hepatectomy was performed. The intraoperative findings were that there was a necrotic friable tumor in the right lobe of the liver with autodemarcation of the right and left lobes. The portal triad was free of tumor.

**Fig 2:**
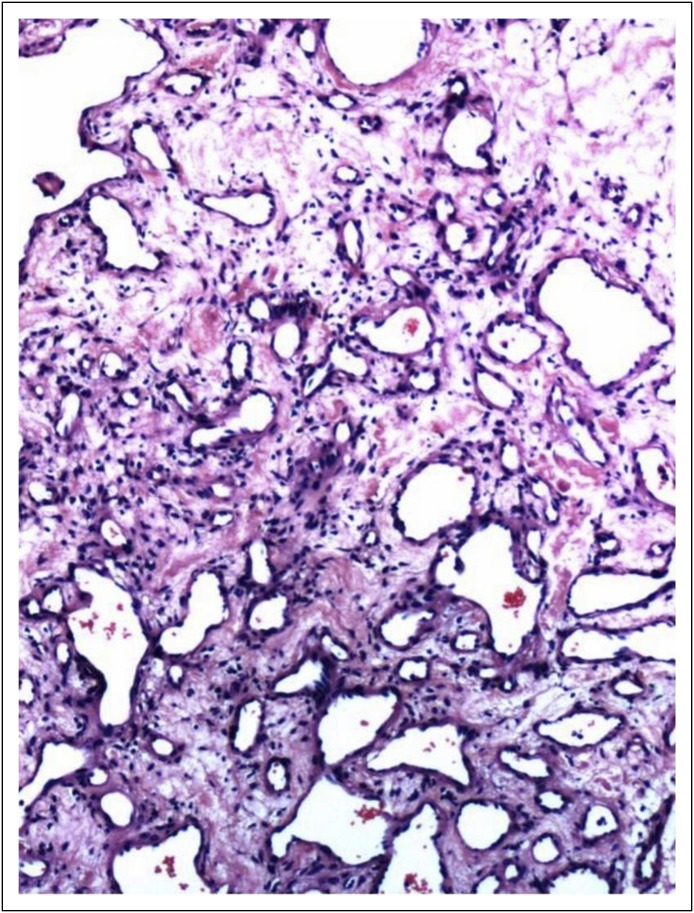
Histopathology images of the surgical specimen. The complete architectural effacement of liver parenchyma is shown, with evidence of tumor composed of numerous blood vessels of both smaller and larger lumen. Extensive areas of fibrosis and hemorrhage were observed. The tumor did not infiltrate the sinusoids. The adjacent liver parenchyma appeared normal. Focal bile duct proliferation was observed.

The postoperative surgically resected specimen was later reported as a hemangioma (Fig 2). Sorafenib was then discontinued. The patient is asymptomatic and regular clinical follow-up is ongoing.

## DISCUSSION

Liver hemangiomas are found in approximately 7% of the general population. Most of the time they are asymptomatic, and they affect women more than men. Approximately 40% of giant hepatic hemangiomas (> 4 cm) produce symptoms that include early satiety, abdominal pain, and sometimes nausea and anorexia.

Hepatic hemangiomas on a dynamic CT scan show an initial intense peripheral nodular enhancement with a gradual central fill-in. Because of this typical radiologic appearance, hemangiomas can be differentiated from other tumors. Sometimes hemangiomas can present with atypical enhancement patterns because of the presence of intralesional nonenhanced thrombosis, degenerated, fibrotic, or calcified components.^6^ Atypical enhancement patterns of hemangiomas are also observed because of the changes in vascularity.^7^ An atypical hemangioma can sometimes mimic a malignant hepatic tumor and cause diagnostic confusion.

In the case reported herein, a patient presented with atypical radiologic features and was initially diagnosed with HCC, which after surgery was found to be a hemangioma.

Treatment options for symptomatic hemangiomas include resection, ligation of the hepatic artery, radiation therapy, and rarely, in selected cases, liver transplantation can be performed.^8-10^ Chemoembolization was also reported to be an effective treatment option for giant hepatic hemangioma.^11^ However, it is still not a well-established option because in some cases it causes an increase in tumor mass.^12^ High morbidity rates of 10% to 27% and a mortality rate of 2% after resection or enucleation of the hepatic hemangioma have been reported in various studies.^13,14^

Propranolol (a nonselective beta blocker) is an effective treatment option for proliferative hemangioma.^15^ It works on the growing hemangioma by the following three mechanisms: vasoconstriction, induction of apoptosis, and downregulation of angiogenic factors.^15,16^ Furthermore, propranolol exerts an inhibitory effect on matrix metalloproteinase 9, which is involved in upregulation of the angiogenesis process. It has been used for the treatment of infantile hemangioma, with effects ranging from significant reduction in size to complete resolution of the hepatic hemangioma.^17^

Some studies have shown activity of bevacizumab in hepatic hemangiomas.^18^ Although the pathogenesis of cavernous hemangioma is not known, it is hypothesized that they are formed because of upregulation of angiogenic factors (eg, vascular endothelial growth factor [VEGF]) and downregulation of antiangiogenesis.^19,20^ Compared with hepatic sinusoidal epithelial cells, VEGF-A is overexpressed in cavernous hepatic hemangiomas and leads to increased angiogenesis.^11^ Bevacizumab exerts its effect by blocking VEGF-A.^18^

In this case report, after sorafenib was administered to a patient with a diagnosis of HCC, there was an impressive response in size of the hepatic mass. Sorafenib is a multikinase inhibitor that works by inhibiting epidermal growth factor receptor; VEGF receptors 1, 2, and 3; and platelet-derived growth factor receptor-β.^21^ Sorafenib is effective against hepatocellular carcinoma^22-24^ and soft tissue sarcomas.^25^ This drug has also been tried for the treatment of patients with vascular tumors, including angiosarcoma, epithelioid hemangioendothelioma, and hemangiopericytoma/solitary fibrous tumor.^26^

In conclusion, we report an unusual case of a woman with a giant hepatic hemangioma, for whom sorafenib was found to be beneficial with tolerable adverse effects. Additional clinical trials should be performed to prove the efficacy of sorafenib in adult hepatic hemangiomas.
